# *ZC3H7A/B*::*BCOR* fusion fibromyxoid sarcoma of soft tissue: an emerging aggressive sarcoma overlapping with malignant ossifying fibromyxoid tumors

**DOI:** 10.1007/s00428-025-04177-4

**Published:** 2025-07-24

**Authors:** Bharat Rekhi, Altan Kavuncuoglu, Nasir Ud Din, Sameer Rastogi, Ali Abdelsatir, Robert Stoehr, Abbas Agaimy, Kemal Kosemehmetoglu

**Affiliations:** 1 Department of Pathology, Tata Memorial Hospital, Parel, Mumbai, Maharashtra India 400012; 2 Division of Molecular Pathology and Translational Medicine, Tata Memorial Hospital, Parel, Mumbai, Maharashtra India; 3 Homi Bhabha National Institute, University (HBNI), Parel, Mumbai, Maharashtra India; 4 Institute of Pathology, University Hospital Erlangen (UKER), Friedrich-Alexander University Erlangen-Nuremberg (FAU), Erlangen, Germany; 5 Department of Pathology and Laboratory Medicine, University of Miami Miller School of Medicine, Miami, FL USA; 6 Department of Medical Oncology, All Indian Institute of Medical Sciences, Ansari Nagar, New Delhi, 110029 India; 7 HistoCenter, Khartoum, Sudan; 8 Comprehensive Cancer Center Erlangen-EMN (CCC, ER-EMN), Erlangen, Germany; 9 Department of Pathology, Hacettepe University Faculty of Medicine, Ankara, Turkey

**Keywords:** Soft tissue tumors, Classification, Molecular profiling, NGS, Targeted therapy, WHO, Translocation

## Abstract

*BCOR*-rearranged sarcomas constitute ultra-rare tumors. Among these, *ZC3H7A/B::BCOR* sarcomas are less common and are primarily reported as a subset of high-grade endometrial stromal sarcomas, as well as in the spectrum of malignant ossifying fibromyxoid tumors (OFMTs). Herein, we present the clinicopathological, immunohistochemical, and molecular profiles of seven soft tissue tumors exhibiting *ZC3H7A/B::BCOR* fusions. The patient’s age ranged from 13 to 65 years (median = 38). Locations were neck (2) and one case each in the paraspinal region, scalp, gluteal region, chest wall, and thigh. Histologically, the tumors were composed of round to polygonal or spindle-shaped cells with a variable amount of fibromyxoid stroma, lacking bone shell or ossification, leading to a range of initial differential diagnoses. Immunohistochemically, the tumor cells were positive for S100 (5/6), cyclin D1 (2/3), SATB2 (2/3), BCOR (2/4), and TLE1 (1/3) while negative for MUC4 (0/6), keratin (0/5), EMA (0/4), desmin (0/6), CD34 (0/6), SMA (0/5), SOX10 (0/5), and melanoma cocktail (0/2). Targeted RNA sequencing revealed *ZC3H7B::BCOR* fusions in six tumors (four with *ZC3H7Bex10::BCORex6* and one each *ZC3H7Bex12::BCORex7* and *ZC3H7Bex12::BCORex6*). One tumor revealed a *ZC3H7Aex10::BCORex6* fusion. All seven tumors were resected, mostly with clear margins (5/7), including two patients who received adjuvant therapy. Three of four patients with available follow-up (mean = 45 months) died of disease, while one patient was alive with multiple bone metastases. This series comprises seven additional *ZC3H7A/B::BCOR* soft tissue sarcomas associated with aggressive clinical outcomes. Whether this aggressive sarcoma represents a molecular subtype of malignant OFMT or a genetic variant of *BCOR*-rearranged sarcomas remains to be further verified.

## Introduction

*BCOR* gene alterations are uncommon genetic drivers reported in sarcomas and high-grade glial neoplasms in pediatric patients and young adults [1]. Among sarcomas, these alterations characterize *BCOR::CCNB3* fusion sarcoma, *BCOR*-internal tandem duplication (ITD)-positive primitive myxoid mesenchymal tumors of infancy (PMMTI), clear cell sarcoma of the kidney, subset of malignant ossifying fibromyxoid tumor (OFMT), and, less frequently, sarcomas with *BCOR::MAML3* and *ZC3H7B::BCOR* fusions. [2–10] *BCOR*—ITD and *ZC3H7B::BCOR* sarcomas also occur as a molecular subset of high-grade endometrial stromal sarcoma (ESS) in the female genital tract [8, 11].

The *BCOR* gene, located at Xp11.4, encodes a ubiquitously expressed transcriptional repressor that combines with the BCL6 oncoprotein and with several histone modifying enzymes, leading to direct gene silencing by a characteristic combination of epigenetic modifications [2, 12, 13]. BCOR regulates early development, hematopoiesis, and mesenchymal stem cell function [14, 15] *ZC3H7B*, also known as RoXaN or ROXAN1, is located on chromosome 22 and encodes a protein that contains a tetratricopeptide repeat domain that interacts with the rotavirus non-structural protein NSP3. Its paralog, *ZC3H7A*, located on chromosome 16, enables miRNA binding activity and processing and acts upstream with a positive effect on post-transcriptional regulation of gene expression. Both ZC3H7 proteins recognize ATA(A/T) motifs in the apical loops of the hairpins of MIR7-1, MIR16-2, and MIR29A precursors and regulate MIR7-1 biogenesis [16].

While *ZC3H7B::BCOR* fusions have been reported in a rare subset of OFMT, it remains currently unclear whether *ZC3H7B::BCOR* fusion-associated soft tissue neoplasms belong to the spectrum of OFMT and *BCOR*-rearranged sarcomas (as a molecular subtype), represent a distinctive category of fusion-driven fibromyxoid sarcomas or form a heterogeneous category comprising both. We herein present detailed clinicopathological and molecular characteristics of seven soft tissue-based neoplasms characterized by variable morphological resemblance to OFMT, but lacking ossification, and carrying *ZC3H7B::BCOR* fusions and review seven previously reported cases.

## Material and methods

The cases were identified in the routine and authors’ consultation files. None of the cases were reported before. Given their consultative nature, immunohistochemistry (IHC) was performed in different laboratories and the stains varied, based on tissue availability and the initial differential diagnostic consideration (details of the staining protocols and antibody sources are available upon request).

### Targeted RNA sequencing

For fusion testing in Case 1, tumor DNA was extracted from the FFPE block and used to perform targeted gene capture using a custom hybrid capture kit. The quality control (QC) passed libraries were sequenced to a minimum depth of 250 × on a validated Illumina sequencing platform. The sequences were processed using a customized and validated analysis pipeline designed to accurately detect all the classes of genomic alterations (single nucleotide variants, InDels, and fusions). The variants were annotated using an in-house annotation pipeline. The reportable genomic alterations and fusions were prioritized, classified, and reported based on the Association of Molecular Pathologists-American Society of Clinical Oncology-College of American Pathologists (AMP-ASCO-CAP) guidelines [17] and the National Comprehensive Cancer Network® (NCCN) guidelines. Fusion analysis for Case 3 was performed by Foundation One. For cases 2 and 4–7, RNA was isolated from formalin-fixed paraffin-embedded (FFPE) tissue sections using RNeasy FFPE Kit of Qiagen (Hilden, Germany) and quantified spectrophotometrically using NanoDrop-1000 (Waltham, USA). Molecular analysis was performed using the TruSight RNA Fusion panel (Illumina, Inc., San Diego, CA, USA) with 500 ng RNA as input according to the manufacturer’s protocol. Libraries were sequenced on a MiSeq (Illumina, Inc., San Diego, CA, USA) with > 3 million reads per case, and sequences were analyzed using the RNA-Seq Alignment workflow, version 2.0.1 (Illumina, Inc., San Diego, CA, USA). The Integrative Genomics Viewer (IGV), version 2.2.13 (Broad Institute), was used for data visualization.

## Results

### Clinical findings

**Fig. 1:**
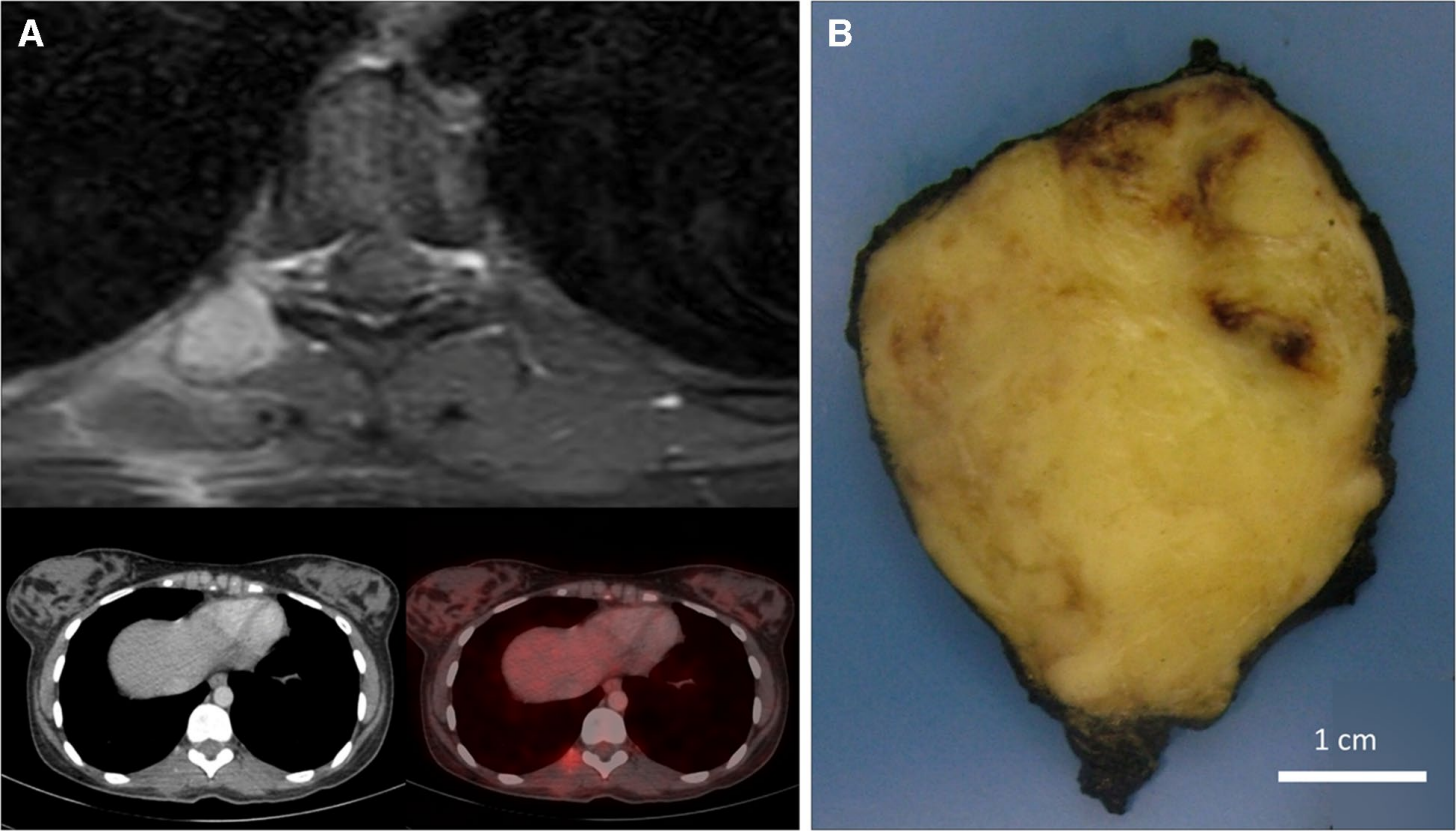
**A** Case 1. Magnetic resonance imaging (MRI) showing a 7.5-cm diameter soft tissue tumor in the right paraspinal region and corresponding positron emission tomography-computed tomography (PET-CT) images. **B** Case 2. Marginal excision of a 6-cm diameter soft tissue tumor with homogeneous myxoid cut-surface with foci of hemorrhage. Note the presence of small nodular extensions in pseudocapsule

The clinicopathological findings, including those of previously reported cases, are summarized in Tables 1 and 2. Tumors occurred in 4 male and three female patients (M: F ratio, 1.25:1), with patients’ ages ranging from 13 to 65 years (median = 38). The tumors were located in the neck (2) and one case each in the paraspinal region, scalp, gluteal region, chest wall, and thigh (Fig. 1A). The tumor size, known in 4 patients, ranged from 2.5 to 7.5 cm (Fig. 1B). All seven tumors were resected, mostly with clear margins (5/7), including two patients (Patients 1 and 2) who were offered adjuvant chemotherapy ± radiotherapy. Three of four patients with available follow-up (mean = 45 months) died of disease, while a single patient (Patient 1) is alive with disease with multiple skeletal metastases at 15 months of follow-up.

**Table 1.** Clinical data including treatment and outcomes of 14 *ZC3H7A/B::BCOR* fusion-positive soft tissue sarcomas including 7 previously reported cases

Authors, year	Age/gender	Site	Tumor size (cm)	Treatment	Outcome
Antonescu et al., [4] 2014	55/M	Thigh	NA	NA	NA
Specht et al., [5] 2016	57/F	Pelvis. Deep	11	Partial resection	NA
Specht et al., [5] 2016	42/M	Arm	6.3	Chemotherapy (VAC), RT	Recurrence. Mets. (Bone and pancreas). DOD (60 mos.)
Watson et al., [35] 2018	53/M	Abdominal wall	NA	NA	NA
Yoshida et al., [23] 2019	45/F	Chest wall. Deep	3	Surgical excision (incomplete), adjuvant RT Resection of metastatic nodules	Lung mets. (1 mo.) AWD (42 mos.)
Linos et al., [21] 2020	49/M	Shoulder. Subcutaneous/deep	8	Wide excision with free margins	No evidence of disease (4 mos.)
Soukup et al., [22] 2023	61/M	Hip. Subcutaneous/deep	4	Incomplete excision, followed by complete excision	Recurrence (36 mos.) Died of unrelated disease (47 mos.)
Present cases (*n* = 7), 2025					
Case #1	22/F	R paraspinal/deep. Radiologically, heterogeneous mass, D9-D12. Impression: Koch’s tuberculosis	7.5	Surgical excision, adjuvant RT, CT (3 cycles, ifosfamide, adriamycin and mesna)	Multiple skeletal mets AWD (15 mos.)
Case #2	45/M	Neck	6	Excision	Pleural mets. DOD (84 mos.)
Case #3	13/M	Gluteal	4.5	Complete resection + adjuvant CT	Lung mets. DOD (36 mos.)
Case #4	24/F	Scalp	NA	Complete resection	NA
Case #5	38/M	Thigh	NA	Complete resection	NA
Case #6	65/M	R chest wall	NA	Complete resection	NA
Case #7	54/F	Back of the neck	2.5	Complete excision	DOD (duration not known)

*M* male, *F* female, *NA* not available, + positive, − negative, *VAC* vincristine, adriamycin, cyclophosphamide, *RT* radiation therapy, *CT* chemotherapy, *Mets* metastasis, *DOD* died of disease, *AWD* alive with disease, *mo.* month

**Table 2.** Histopathological diagnosis, immunohistochemical profile and molecular findings of 14 *ZC3H7A/B::BCOR* fusion-positive soft tissue sarcomas including 7 previously reported cases

Authors, year	Submitted diagnosis	Histopathological diagnosis	Positive immunostains	Negative immunostains	Molecular findings
Antonescu et al., [4] 2014	**-**	Malignant ossifying fibromyxoid tumor (OFMT) with ossification	**-**	S100P, desmin	***ZC3H7Bex10::BCORex7***
Specht et al., [5] 2016	-	Small blue round cell tumor lacking *EWSR1, FUS, SYT* and *CIC* gene rearrangements	CD99	Keratin, S100P, desmin, WT1	***ZC3H7B::BCOR***
Specht et al., [5] 2016	-	Small blue round cell tumor lacking *EWSR1, FUS, SYT* and *CIC* gene rearrangements	CD99	Keratin, S100P	***ZC3H7B::BCOR***
Watson et al., [35] 2018	-	Ewing-like sarcoma	NA	NA	***ZC3H7B::BCOR***
Yoshida et al., [23] 2019	Desmoid tumor	-	BCOR, SATB2, CD10	AE1/AE3, EMA, S100P, SMA, desmin, myogenin, CD34, D2-40, ER, CCNB3, INI1 retained, H3K27me3 retained	***ZC3H7Bex12::BCORex7***
Linos et al., [21] 2020	-	OFMT	S100P, CD10, PR, claudin-1, Ki67-15–20%	BCOR, desmin, ER, CK, MUC4, SMA, EMA, CD34, STAT6, H3K27me3 retained	***BCOR* FISH positive** ***ZC3H7Bex12::BCORex7***
Soukup et al., [22] 2023	-	Low-grade fibromyxoid sarcoma	-	BCOR, CD99, EMA, S100P, AE1/AE3, MUC4, desmin, SOX10, SOX11, CD34, WT1, INSM1, GFAP, CD57	***ZC3H7Bex10::BCORex7***
Present cases (*n* = 7), 2025					
Case #1	Extraskeletal myxoid chondrosarcoma	Sarcoma, grade 2 with myxoid stroma	CyclinD1, SATB2	S100P, SOX10, BCOR, MUC4, AE1/AE3, GFAP, EMA, desmin, p63-, CD34, TLE1, INSM1, synaptophysin	***BCOR* FISH positive** ***ZC3H7Bex12::BCORex7***
Case #2	Myxoid liposarcoma	Malignant OFMT	S100P	BCOR, cyclinD1, SATB2, SOX10, TLE1, CD34, panTRK, EMA, AE1/AE3, desmin, SMA, myoD1, MUC4, NUT, β-catenin, H3k27me3 retained	***ZC3H7Bex10::BCORex6***
Case #3	-	OFMT	S100P-Focal, BCOR-Focal, SATB2, CyclinD1, TLE1-Multifocal	AE1/AE3, SMA, desmin, myogenin, synaptophysin	***ZC3H7Bex12::BCORex6***
Case #4	-	Fibromyxoid tumor unclassified	BCOR	Desmin, EMA, MUC4, CD34, INI1- retained	***ZC3H7Bex10::BCORex6***
Case #5	-	OFMT-like neoplasm (non-ossifying)	S100P-Focal, CD56	Desmin, SMA, CD34, AE1/AE3, MUC4, p63, CD117, SOX10 and Pan-Melanoma	***ZC3H7Bex10::BCORex6***
Case #6	-	High-grade sarcoma with inflammatory and myxoid features	S100P-Focal	Desmin, SMA, MUC4, ALK, SOX10, CD34, Pan-melanoma	***ZC3H7Aex10::BCORex6***
Case #7	-	Translocation-related sarcoma (*BCOR*-altered sarcoma?)	S100P	AE1/AE3, SOX10, SMA, STAT6, EMA, ERG, MUC4, desmin, SS18-SSX, MDM2, CDK4, CD34	***EWSR1* FISH negative** ***ZC3H7Bex10::BCORex6***

*NA* not available

### Pathological findings

**Fig. 2:**
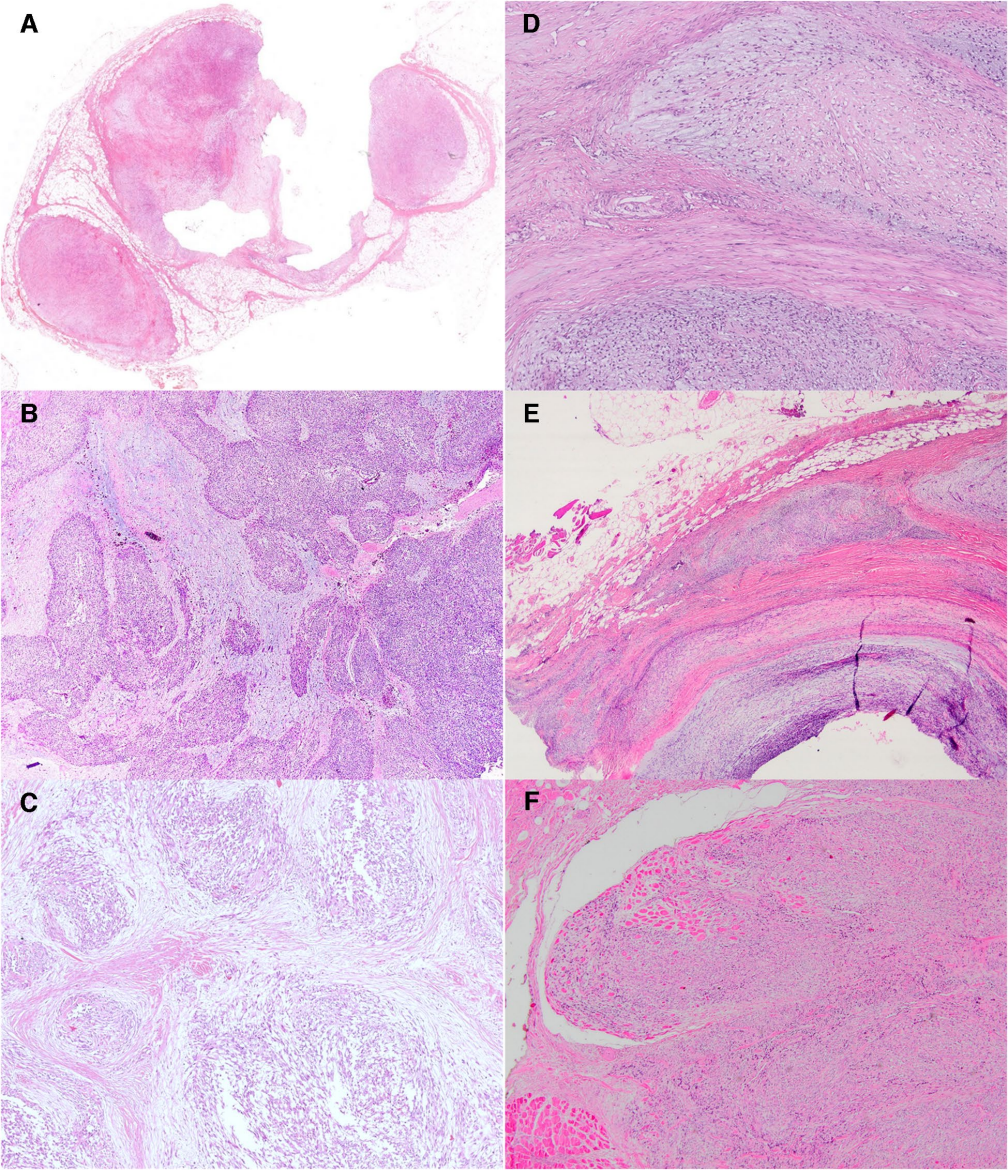
Vague multinodularity formed by alternating hypo- and hypercellular areas (**A**, Case 6; **B**, Case 3; and **C**, Case 5). Low-power microscopical features include finger-like extensions of neoplastic cells within the surrounding soft tissue without a shell of bone or ossification (**D**, Case 1; **E**, Case 2; and **F**, Case 7)

**Fig. 3:**
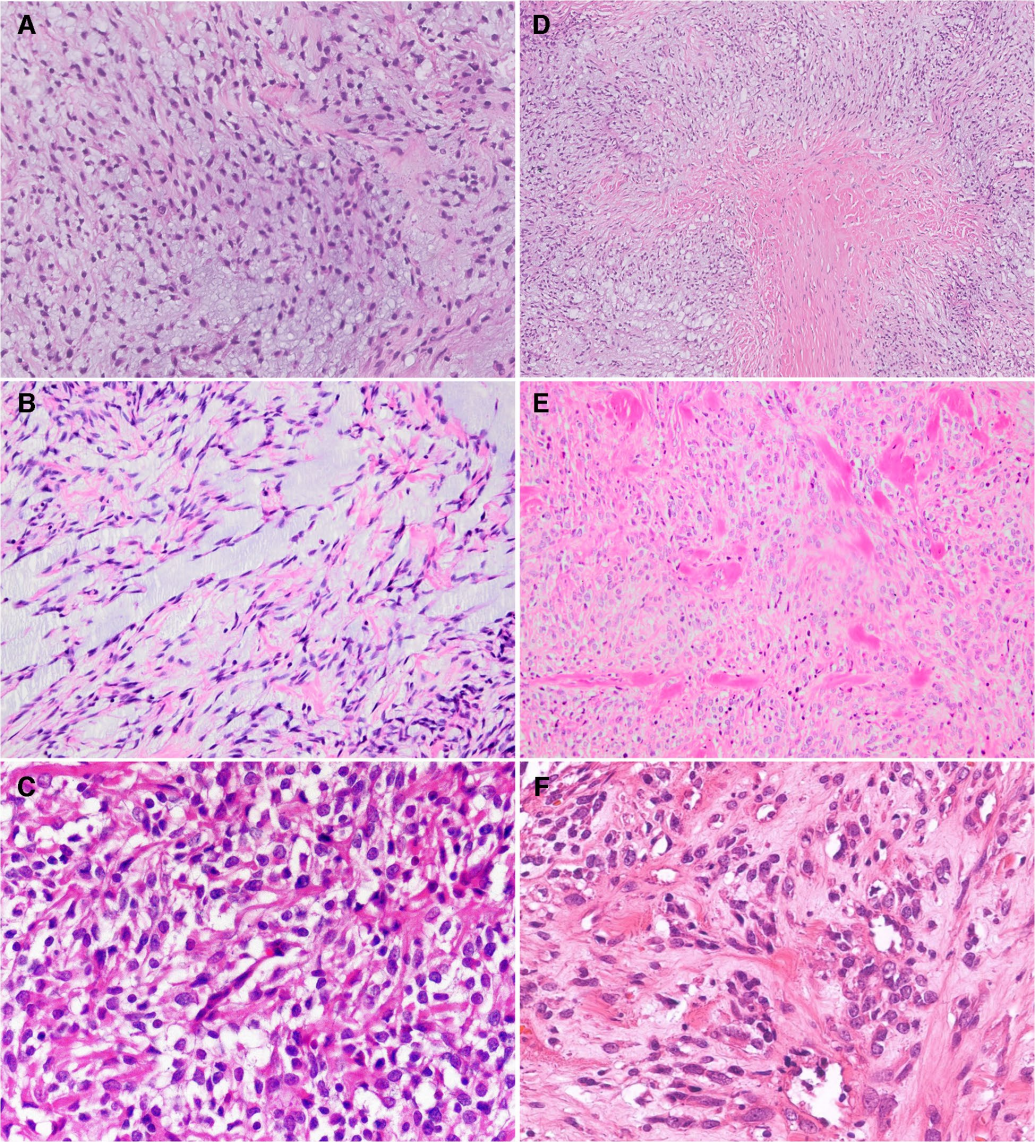
High-power microscopical features: **A** Cellular tumor composed of oval to spindle-shaped cells arranged in a non-descript sheeted pattern with a prominent myxoid stroma, including a focal area reminiscent of an ossifying fibromyxoid tumor (Case 1). **B** Hypocellular myxoid areas and cord-like pattern resembling extraskeletal myxoid chondrosarcoma (Case 2). **C** Hypercellular areas comprising small round cells with inconspicuous nucleoli (Case 3). **D**, **E**, and **F** Different patterns of stromal collagenization (**D**, Case 1; **E**, Case 7; and **F**, Case 4). Note the presence of accompanying inflammatory cells (**C**, **E**, and **F**)

Histopathologically, the tumors mainly were multinodular (Fig. 2A–C), composed of oval/epithelioid to spindle-shaped (Fig. 3A) and round to epithelioid (tumors 3, 5, and 7, Fig. 3C) cells, primarily arranged in fascicles, sheets and focal cord-like pattern (Fig. 3B), within a variable fibromyxoid stroma. Necrosis was present in only one tumor (case 7). Mitotic figures ranged from 0–16/10 high-power field. A striking morphological feature was finger-like extensions in the surrounding soft tissues, seen in four tumors (Fig. 2D–F) and variable collagen fibers around the neoplastic cells (Fig. 3D–F). There was no ossification or bone shell formation in any tumors, except small foci of bone in Case 5. There were interspersed chronic inflammatory cells, including lymphocytes and mast cells, seen in four tumors (Fig. 3C, E, and F).

**Fig. 4:**
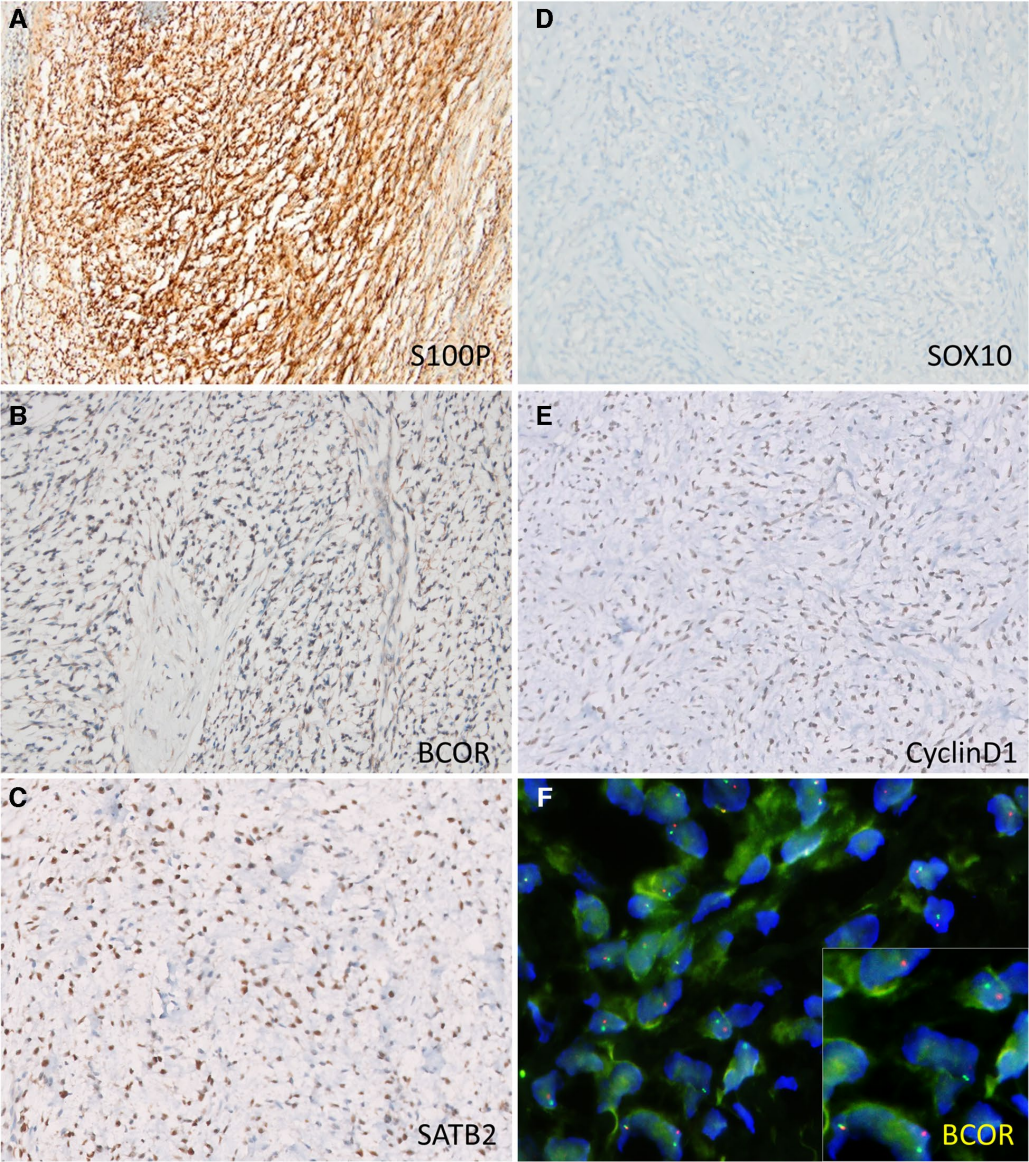
Immunohistochemical results: neoplastic cells show patchy or diffuse S100P (**A**, Case 2) expression, while SOX10 is negative (**B**, Case 7). BCOR (**C**, Case 3), CyclinD1 (**D**, Case 1), and SATB2 (**E**, Case 1) are usually positive. **F** Tumor cells displaying several red-green “split” signals indicative of *BCOR* rearrangement by FISH

Immunohistochemically, the tumor cells were positive for S100 (5/6), cyclin D1 (2/3), SATB2 (2/3), BCOR (2/4), and TLE1 (1/3) while negative for MUC4 (0/6), keratin (0/5), EMA (0/4), desmin (0/6), CD34 (0/6), SMA (0/5), SOX10 (0/5), and pan melanoma (0/2) (Fig. 4).

### Molecular findings

**Fig. 5:**
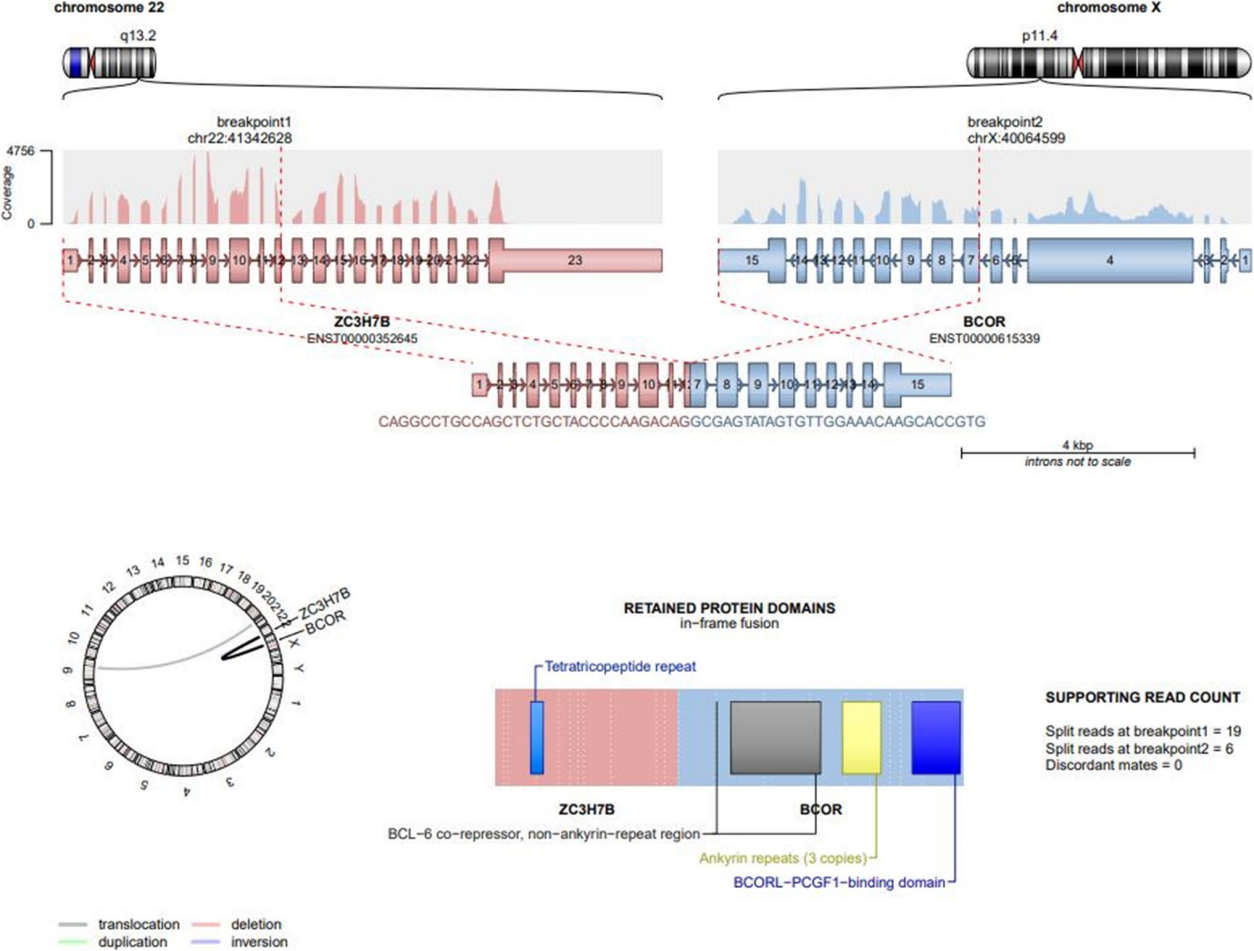
Representative schematic illustration of the ZC3H7B(exon12)::BCOR(exon7) fusion from Case 1. This figure illustrates the intricate genetic interactions between chromosomes 22 and X, highlighting the breakpoints, gene sequences, and retained protein domains

On next-generation sequencing (NGS), six tumors revealed *ZC3H7B::BCOR* fusion, including *ZC3H7Bex10::BCORex6* (*n* = 4), *ZC3H7Bex12::BCORex7* (*n* = 1), *ZC3H7Bex12::BCORex6* (*n* = 1), and one tumor revealed *ZC3H7Aex10::BCORex6* fusion (Fig. 5). A single tumor (Case 1) was also tested for *BCOR* gene rearrangement by fluorescence in situ hybridization, which was positive, with 75% of the tumor cell nuclei displaying red-green “split” signals (Fig. 4F).

## Discussion

*ZC3H7B::BCOR* or, rarely, *BCOR::ZC3H7A*-rearranged sarcomas are rare, constituting 5% of all *BCOR*-altered sarcomas [18]. Most are reported as a subset of uterine high-grade ESSs or ossifying fibromyxoid tumors (OFMT), including malignant OFMTs [19–21]. The present series constitutes seven cases of primary *ZC3H7A/B::BCOR* soft tissue sarcomas occurring in various anatomic sites in adults with a mean age of 38 years and a slight predilection for males [22]. In a series of 75 small blue round cell tumors lacking *EWSR1, FUS, SYT, CIC*, and *BCOR::CCNB3*, Specht et al. [5] attempted to detect potential recurrent *BCOR* gene rearrangements outside the typical X-chromosomal inversion. They identified 2 cases, each of a *BCOR::MAML3* and *ZC3H7B::BCOR* sarcoma. In another study, Yoshida et al. [23] reported a single *ZC3H7Bex7::BCORex7* out of 7 *BCOR*-rearranged sarcomas in an adult patient. To date, only seven such tumors are reported in the soft tissues, mainly in adult patients with an almost equal gender predisposition (Table 1). These seven previously reported tumors were initially diagnosed as malignant OFMT, so-called “Ewing-like” sarcoma, desmoid tumor, and low-grade fibromyxoid sarcoma, respectively. Among 14 reported patients, including seven from the present study, nine males and five females were affected, with a median age of 47 and a mean age of 44.5 [4, 5, 21, 23].

Histopathologically, various tumors exhibiting *BCOR* gene rearrangement display oval to spindle cells, thin-walled blood vessels, and a significant amount of myxoid areas, as noted in all tumors of the present study [2–11, 18–22]. While a single tumor, each in the present series was referred with a diagnosis of extraskeletal myxoid chondrosarcoma and myxoid liposarcoma, respectively, OFMT, including malignant OFMT, was the most frequent initial diagnosis, based on morphology and immunostains, followed by sarcoma with myxoid features/stroma (Table 2). The primary question is whether these tumors represent malignant OFMTs or a variant of the *BCOR*-rearranged sarcoma family as an independent neoplasm. In an extensive series of OFMT, Folpe et al. [24] reported a median age of 49 years, with most tumors occurring as subcutaneous to deep soft tissue masses, similar to the tumors in the current study. They observed ossification and S100 positivity in 63% and 60% of tumors, respectively. Pankeratin (10%) and desmin (13%) were expressed infrequently. In another study; a partial shell of bone/ossification was reported in 60% of OFMTs, with a high frequency in typical and a lesser frequency in malignant OFMTs [25]. In the same study, S100 positivity was observed in 73% of OFMTs, which was higher in typical than malignant OFMTs. Subsequent studies showed *PHF1* gene rearrangement in 50% of OFMTs, including typical, atypical, and malignant subtypes [26, 27]. Gebre-Medhin et al. [27] observed *PHF1* rearrangement in 7/13 (54%) OFMTs, most frequently in typical, followed by atypical and least in malignant OFMTs. Antonescu et al.[4] described two morphologically convincing malignant OFMTs with *BCOR* rearrangements — one of which involved Z*C3H7B* as a partner gene — and both cases were classified as malignant but with typical ossification, which occurred in the thigh of elderly males (55 and 76 years old), and both tumors lacked S100 and desmin expression. In the present study, 6/7 tumors lacked ossification, and all seven tumors lacked *PHF1* rearrangement, despite variable S100 positivity in 5 of 6 tumors. These features are more commonly associated with an OFMT and constitute its significant diagnostic criteria in the current WHO classification [4, 19, 28–30]. None of the tumors showed desmin expression. At the same time, multinodularity, finger-like extension, fibromyxoid stroma, and focal cord-like arrangement of tumor cells with S100 positivity in some of the tumors overlap with OFMT.

BCOR immunostain is invariably positive in most *BCOR-*rearranged sarcomas, including the *BCOR::CCNB3*, *BCOR::MAML3*, *BCOR*-ITD sarcomas, and also in certain non-*BCOR*-rearranged sarcomas, such as *YWHAE::NUTM2B* sarcomas [31]. However, BCOR was reportedly positive in 1/2 *BCOR::ZC3H7B* (positive in *BCORex7::ZC3H7Bex11* and negative in *BCORex6::ZC3H7Bex11)* and in 2 *ZC3H7B::BCOR*-rearranged high-grade ESSs (with *ZC3H7Bex6::BCORex14* and *ZC3H7Bex10::BCORex17* fusions) [19]. We observed BCOR positivity in two of four tested tumors. BCOR negativity in the first two tumors in this study created a challenge in the diagnosis of a *BCOR*-rearranged sarcoma. Soukup et al. [22] observed BCOR negativity in a *ZC3H7B::BCOR* sarcoma. Although highly sensitive, BCOR immunostain might not be the perfect surrogate for a *BCOR*-rearranged sarcoma, as this marker is increasingly reported in diverse sarcomas lacking *BCOR* alterations, and it can be absent in *BCOR*-rearranged sarcomas. Cyclin D1, SATB2, TLE1, and CD10 are other immunostains that are not specific but helpful in considering a *BCOR* rearranged sarcoma, as noted in Cases 1 and 3 in this series. TLE1 was positive in 1/3 of tumors tested [10, 11, 23].

Apart from OFMT, various other differential diagnoses in the current cases included low-grade fibromyxoid sarcoma (LGFMS), considered in six tumors, and other soft tissue sarcomas with a variable amount of myxoid differentiation. MUC4 negativity ruled out LGFMS, as previously reported.[22] Absence of tumor cords and trabeculae, along with NSE and INSM1 immunonegativity, made a diagnosis of EMC less likely [32]. Absent staining for epithelial markers, S100, and GFAP ruled out myoepithelial neoplasm [33]. Lack of a network of thin-walled blood vessels and lipoblasts ruled out a myxoid liposarcoma. CD34 negativity was against DFSP, while SOX10 negativity and retained H3K27me3 immunostaining argued against an MPNST. Lack of CD34 and panTRK preclude an *NTRK*-rearranged mesenchymal tumor. PanTRK immunopositivity is reported in rare tumors with overlapping features designated as malignant OFMTs. [34] The presence of round cells can lead to the consideration of “Ewing-like” sarcoma. [35] Overall, all these differential diagnostic considerations listed above and discussed in brief are indeed excluded by the lack of their defining gene fusions that were not detected by NGS in any of the cases.

In terms of treatment, most of these documented tumors were primarily managed with surgical resection with or without adjuvant radiation therapy, as noted in our study. The best choice of chemotherapy for these sarcomas is not well defined yet, due to the rarity of the disease and paucity of available data. The outcomes seem unfavorable, including recurrences and metastasis, as similarly observed in our study [5, 21, 22]. Given that 3 out of 4 patients with available follow-up in the present study died of disease and another patient developed metastasis, these tumors are aggressive sarcomas. Overall, among 8/14 documented tumors with available follow-up, including the present cases, two patients developed tumor recurrences and five developed metastasis. Finally, 4/8 patients died of disease, and 2 were alive with disease, underscoring their aggressive clinical course.

In conclusion, *Z3CH7B/A::BCOR* sarcomas are extremely rare in soft tissues. These tumors may appear deceptively low-grade, have overlapping features with OFMT, or resemble undifferentiated round cell sarcomas, still behaving aggressively in the majority (> 50%) of cases. A morphological approach, based upon certain histopathological features, ruling out their various differentials, supported by appropriate immunostains, especially S100, desmin, and BCOR, seems feasible to further triage these tumors for molecular testing, including NGS. It is essential to know that even though BCOR immunostain is positively expressed in most *BCOR*-rearranged sarcomas, it could be negative in rare variants and can be detected in diverse non-BCOR-altered mesenchymal neoplasms. Accordingly, high-throughput molecular testing in such tumors and FISH for *BCOR* rearrangement seems useful.

Currently, it remains controversial whether *ZC3H7A/B::BCOR*-positive sarcomas constitute another provisional entity of a “fusion-driven” soft tissue sarcoma or a genetic subset/variant in the spectrum of malignant OFMTs or *BCOR*-rearranged sarcomas. To enable better characterization of these rare aggressive sarcomas, we suggest reporting them separately as “*ZC3H7A/B::BCOR* fusion fibromyxoid sarcoma of soft tissue,” until further data and dee functional and epigenetic analyses establish or rule out their presumed relationship to malignant OFMT and other *BCOR*-rearranged sarcomas. Documentation of more such tumors will provide additional insights towards their biological behavior and further optimization of therapeutic approaches for these ultra-rare aggressive sarcomas that do not respond to conventional therapies and are associated with recurrences and metastasis.

## Data Availability

The datasets generated during and/or analyzed during the current study are not publicly available, but are available from the corresponding author upon reasonable request.

## References

[1] Mankuzhy NP, Anderson B, Kumar C, Heider A, Koschmamn C, Mody RJ (2019) BCOR alterations in pediatric and young adult patients with sarcomas and high-grade glial malignancies: a case series. JCO Precis Oncol 3:1–8 35100716 10.1200/PO.19.00116

[2] Pierron G, Tirode F, Lucchesi C, Reynaud S, Ballet S, Cohen-Gogo S, Perrin V, Coindre JM, Delattre O (2012) A new subtype of bone sarcoma defined by BCOR::CCNB3 gene fusion. Nat Genet 44:461–466 22387997 10.1038/ng.1107

[3] Puls F, Niblett A, Marland G, Gaston CL, Douis H, Mangham DC, Sumathi VP, Kindblom LG (2014) BCOR::CCNB3 (Ewing-like) sarcoma: a clinicopathologic analysis of 10 cases, in comparison with conventional Ewing sarcoma. Am J Surg Pathol 38:1307–1318 24805859 10.1097/PAS.0000000000000223

[4] Antonescu CR, Sung YS, Chen CL, Zhang L, Chen HW, Singer S, Agaram NP, Sboner A, Fletcher CD (2014) Novel ZC3H7B-BCOR, MEAF6-PHF1, and EPC1-PHF1 fusions in ossifying fibromyxoid tumors–molecular characterization shows genetic overlap with endometrial stromal sarcoma. Genes Chromosomes Cancer 53:183–193 24285434 10.1002/gcc.22132 PMC4053209

[5] Specht K, Zhang L, Sung YS, Nucci M, Dry S, Vaiyapuri S, Richter GH, Fletcher CD, Antonescu CR (2016) Novel BCOR-MAML3 and ZC3H7B-BCOR gene fusions in undifferentiated small blue round cell sarcomas. Am J Surg Pathol 40:433–442 26752546 10.1097/PAS.0000000000000591 PMC4792719

[6] Argani P, Kao YC, Zhang L, Bacchi C, Matoso A, Alaggio R, Epstein JI, Antonescu CR (2017) Primary renal sarcomas with BCOR::CCNB3 gene fusion: a report of 2 cases showing histologic overlap with clear cell sarcoma of kidney, suggesting further link between BCOR-related sarcomas of the kidney and soft tissues. Am J Surg Pathol 41:1702–1712 28817404 10.1097/PAS.0000000000000926 PMC5680139

[7] Santiago T, Clay MR, Allen SJ, Orr BA (2018) Recurrent BCOR internal tandem duplication and BCOR or BCL6 expression distinguish primitive myxoid mesenchymal tumor of infancy from congenital infantile fibrosarcoma. Mod Pathol 31:374 29430000 10.1038/modpathol.2017.178

[8] Mariño-Enriquez A, Lauria A, Przybyl J, Ng TL, Kowalewska M, Debiec-Rychter M, Ganesan R, Sumathi V, George S, McCluggage WG, Nucci MR, Lee CH, Fletcher JA (2018) BCOR internal tandem duplication in high-grade uterine sarcomas. Am J Surg Pathol 42:335–341 29200103 10.1097/PAS.0000000000000993

[9] Rekhi B, Kembhavi P, Mishra SN, Shetty O, Bajpai J, Puri A (2019) Clinicopathologic features of undifferentiated round cell sarcomas of bone & soft tissues: an attempt to unravel the *BCOR-CCNB3-* & *CIC-DUX4*-positive sarcomas. Indian J Med Res 150:557–574 32048619 10.4103/ijmr.IJMR_2144_18 PMC7038815

[10] Rekhi B, Kosemehmetoglu K, Ergen FB, Vengurlekar V, Rumde R, Shetty O, Guler G (2023) Spectrum of histopathological, immunohistochemical, molecular and radiological features in 12 cases of *BCOR::CCNB3*-positive sarcomas with literature review. Int J Surg Pathol 31:1244–1264 36591870 10.1177/10668969221143467

[11] Lewis N, Soslow R, Delair D, Park K, Murali R, Hollmann T, Davidson B, Micci F, Panagopoulos I, Hoang LN, Arias-Stella JA 3rd, Oliva E, Young RH, Hensley ML, Leitao MM Jr, Hameed M, Benayed R, Ladanyi M, Frosina D, Jungbluth AA, Antonescu CR, Chiang S (2018) ZC3H7B-BCOR high-grade endometrial stromal sarcomas: a report of 17 cases of a newly defined entity. Mod Pathol 31:674–684 29192652 10.1038/modpathol.2017.162

[12] Huynh KD, Fischle W, Verdin E, Bardwell VJ (2000) BCoR, a novel corepressor involved in BCL-6 repression. Genes Dev 14:1810–1823 10898795 PMC316791

[13] Gearhart MD, Corcoran CM, Wamstad JA, Bardwell VJ (2006) Polycomb group and SCF ubiquitin ligases are found in a novel BCOR complex recruited to BCL6 targets. Mol Cell Biol 26:6880–6889 16943429 10.1128/MCB.00630-06 PMC1592854

[14] Wamstad JA, Corcoran CM, Keating AM, Bardwell VJ (2008) Role of the transcriptional corepressor BCOR in embryonic stem cell differentiation and early embryonic development. PLoS ONE 3:e2814 18795143 10.1371/journal.pone.0002814 PMC2535898

[15] Fan Z, Yamaza T, Lee JS, Yu J, Wang S, Fan G, Shi S, Wang CY (2009) BCOR regulates mesenchymal stem cell function by epigenetic mechanisms. Nat Cell Biol 11:1002–1009 19578371 10.1038/ncb1913 PMC2752141

[16] Treiber T, Treiber N, Plessmann U, Harlander S, Daiß JL, Eichner N, Lehmann G, Schall K, Urlaub H, Meister G (2017) A compendium of RNA-binding proteins that regulate MicroRNA biogenesis. Mol Cell 66:270–284 28431233 10.1016/j.molcel.2017.03.014

[17] Li MM, Datto M, Duncavage EJ, Kulkarni S, Lindeman NI, Roy S, Tsimberidou AM, Vnencak-Jones CL, Wolff DJ, Younes A, Nikiforova MN (2017) Standards and guidelines for the interpretation and reporting of sequence variants in cancer: a joint consensus recommendation of the Association for Molecular Pathology, American Society of Clinical Oncology, and College of American Pathologists. J Mol Diagn 19:4–23 27993330 10.1016/j.jmoldx.2016.10.002 PMC5707196

[18] Kyriazoglou A, Bagos P (2021) Meta-analysis of BCOR rearranged sarcomas: challenging the therapeutic approach. Acta Oncol 60:721–726 33630701 10.1080/0284186X.2021.1890818

[19] Chiang S, Lee CH, Stewart CJR, Oliva E, Hoang LN, Ali RH, Hensley ML, Arias-Stella JA 3rd, Frosina D, Jungbluth AA, Benayed R, Ladanyi M, Hameed M, Wang L, Kao YC, Antonescu CR, Soslow RA (2017) BCOR is a robust diagnostic immunohistochemical marker of genetically diverse high-grade endometrial stromal sarcoma, including tumors exhibiting variant morphology. Mod Pathol 30:1251–1261 28621321 10.1038/modpathol.2017.42 PMC5916794

[20] Mansor S, Kuick CH, Lim SL, Quek R, Wong APC, Lim-Tan SK, Lim TYK, Chang KTE (2019) ZC3H7B-BCOR-rearranged endometrial stromal sarcomas: a distinct subset merits its own classification? Int J Gynecol Pathol 38:420–425 29901520 10.1097/PGP.0000000000000523

[21] Linos K, Kerr DA, Baker M, Wong S, Henderson E, Sumegi J, Bridge JA (2020) Superficial malignant ossifying fibromyxoid tumors harboring the rare and recently described ZC3H7B-BCOR and PHF1-TFE3 fusions. J Cutan Pathol 47:934–945 32352579 10.1111/cup.13728

[22] Soukup J, Valtr O, Brtkova J, Zoul Z, Staniczkova-Zambo I, Hojny J, Kamaradova K (2023) Soft tissue sarcoma with ZC3H7B::BCOR fusion in a male mimicking low-grade fibromyxoid sarcoma - a case report. Pathol Res Pract 251:154831 37837859 10.1016/j.prp.2023.154831

[23] Yoshida A, Arai Y, Hama N, Chikuta H, Bando Y, Nakano S, Kobayashi E, Shibahara J, Fukuhara H, Komiyama M, Watanabe SI, Tamura K, Kawai A, Shibata T (2020) Expanding the clinicopathologic and molecular spectrum of BCOR-associated sarcomas in adults. Histopathology 76:509–520 31647130 10.1111/his.14023

[24] Folpe AL, Weiss SW (2003) Ossifying fibromyxoid tumor of soft parts: a clinicopathologic study of 70 cases with emphasis on atypical and malignant variants. Am J Surg Pathol 27:421–431 12657926 10.1097/00000478-200304000-00001

[25] Graham RP, Dry S, Li X, Binder S, Bahrami A, Raimondi SC, Dogan A, Chakraborty S, Souchek JJ, Folpe AL (2011) Ossifying fibromyxoid tumor of soft parts: a clinicopathologic, proteomic, and genomic study. Am J Surg Pathol 35:1615–1625 21997683 10.1097/PAS.0b013e3182284a3f PMC3193600

[26] Graham RP, Weiss SW, Sukov WR, Goldblum JR, Billings SD, Dotlic S, Folpe AL (2013) PHF1 rearrangements in ossifying fibromyxoid tumors of soft parts: a fluorescence in situ hybridization study of 41 cases with emphasis on the malignant variant. Am J Surg Pathol 37:1751–1755 23887158 10.1097/PAS.0b013e31829644b4

[27] Gebre-Medhin S, Nord KH, Möller E, Mandahl N, Magnusson L, Nilsson J, Jo VY, Vult von Steyern F, Brosjö O, Larsson O, Domanski HA, Sciot R, Debiec-Rychter M, Fletcher CD, Mertens F (2012) Recurrent rearrangement of the PHF1 gene in ossifying fibromyxoid tumors. Am J Pathol 181:1069–1077 22796436 10.1016/j.ajpath.2012.05.030

[28] Bakiratharajan D, Rekhi B (2016) Ossifying fibromyxoid tumor: an update. Arch Pathol Lab Med 140:371–375 27028395 10.5858/arpa.2014-0590-RS

[29] Cordier F, Van der Meulen J, Loontiens S, Van Roy N, Lapeire L, Willaert W, Ferdinande L, Van de Vijver K, Van Dorpe J, Creytens D (2023) High-grade endometrial stromal sarcoma-like sarcoma in male: does it exist? A case report and review of the literature. Pathol Res Pract 241:154228 36455366 10.1016/j.prp.2022.154228

[30] Endo M, Mertens F, MIettinen M (2020) Ossifying fibromyxoid tumor. Tumors of uncertain differentiation. In: World Health Organization classification of tumors (WHO) editorial board, editors, 5th edn. World Health Organization Classification of Tumors of Soft Tissue and Bone. .Lyon, France: IARC Press , pp 74–6

[31] Kao YC, Sung YS, Zhang L, Jungbluth AA, Huang SC, Argani P, Agaram NP, Zin A, Alaggio R, Antonescu CR (2016) BCOR overexpression is a highly sensitive marker in round cell sarcomas with BCOR genetic abnormalities. Am J Surg Pathol 40:1670–1678 27428733 10.1097/PAS.0000000000000697 PMC5106294

[32] Yoshida A, Makise N, Wakai S, Kawai A, Hiraoka N (2018) INSM1 expression and its diagnostic significance in extraskeletal myxoid chondrosarcoma. Mod Pathol 31:744–752 29327709 10.1038/modpathol.2017.189

[33] Hornick JL, Fletcher CD (2003) Myoepithelial tumors of soft tissue: a clinicopathologic and immunohistochemical study of 101 cases with evaluation of prognostic parameters. Am J Surg Pathol 27:1183–1196 12960802 10.1097/00000478-200309000-00001

[34] Kao YC, Sung YS, Argani P, Swanson D, Alaggio R, Tap W, Wexler L, Dickson BC, Antonescu CR (2020) NTRK3 overexpression in undifferentiated sarcomas with YWHAE and BCOR genetic alterations. Mod Pathol 33:1341–1349 32034283 10.1038/s41379-020-0495-2 PMC7329614

[35] Watson S, Perrin V, Guillemot D, Reynaud S, Coindre JM, Karanian M, Guinebretière JM, Freneaux P, Le Loarer F, Bouvet M, Galmiche-Rolland L, Larousserie F, Longchampt E, Ranchere-Vince D, Pierron G, Delattre O, Tirode F (2018) Transcriptomic definition of molecular subgroups of small round cell sarcomas. J Pathol 245:29–40 29431183 10.1002/path.5053

