# Correction to: ZC3H7A/B:BCOR fusion fbromyxoid sarcoma of soft tissue: an emerging aggressive sarcoma overlapping with malignant ossifying fbromyxoid tumors

**DOI:** 10.1007/s00428-025-04217-z

**Published:** 2025-08-06

**Authors:** Bharat Rekhi, Altan Kavuncuoglu, Nasir Ud Din, Sameer Rastogi, Ali Abdelsatir, Robert Stoehr, Abbas Agaimy, Kemal Kosemehmetoglu

**Affiliations:** 1 Department of Pathology, Tata Memorial Hospital, Parel, Mumbai, Maharashtra India 400012; 2 Division of Molecular Pathology and Translational Medicine, Tata Memorial Hospital, Parel, Mumbai, Maharashtra India; 3 Homi Bhabha National Institute, University (HBNI), Parel, Mumbai, Maharashtra India; 4 Institute of Pathology, University Hospital Erlangen (UKER), Friedrich-Alexander University Erlangen-Nuremberg (FAU), Erlangen, Germany; 5 Department of Pathology and Laboratory Medicine, University of Miami Miller School of Medicine, Miami, FL USA; 6 Department of Medical Oncology, All Indian Institute of Medical Sciences, Ansari Nagar New Delhi, 110029 India; 7 HistoCenter, Khartoum, Sudan; 8 Comprehensive Cancer Center Erlangen-EMN (CCC, ER-EMN), Erlangen, Germany; 9 Department of Pathology, Hacettepe University Faculty of Medicine, Ankara, Turkey

**Correction to** **:** **Virchows Archiv**

10.1007/s00428-025-04177-4

In Table 2, the fusion gene for Cases 2, 5, and 7 is incorrectly listed as *ZC3H7A*. It should be *ZC3H7B*.

The original article has been corrected.

